# Identification of Susceptibility Genes Underlying Bovine Respiratory Disease in Xinjiang Brown Cattle Based on DNA Methylation

**DOI:** 10.3390/ijms25094928

**Published:** 2024-04-30

**Authors:** Hang Cao, Chao Fang, Ling-Ling Liu, Frederic Farnir, Wu-Jun Liu

**Affiliations:** 1 College of Animal Science, Xinjiang Agricultural University, Urumqi 830052, China; y77996699@126.com (H.C.); linglingliu1988@xjau.edu.cn (L.-L.L.); 2 Faculte de Medecine Veterinaire, Universite de Liege, Quartier Vallee 2, Avenue de Cureghem 6 (B43), 4000 Liege, Belgium; chao.fang@doct.uliege.be

**Keywords:** DNA methylation, calf, bovine respiratory disease, epigenetics

## Abstract

DNA methylation is a form of epigenetic regulation, having pivotal parts in controlling cellular expansion and expression levels within genes. Although blood DNA methylation has been studied in humans and other species, its prominence in cattle is largely unknown. This study aimed to methodically probe the genomic methylation map of Xinjiang brown (XJB) cattle suffering from bovine respiratory disease (BRD), consequently widening cattle blood methylome ranges. Genome-wide DNA methylation profiling of the XJB blood was investigated through whole-genome bisulfite sequencing (WGBS). Many differentially methylated regions (DMRs) obtained by comparing the cases and controls groups were found within the CG, CHG, and CHH (where H is A, T, or C) sequences (16,765, 7502, and 2656, respectively), encompassing 4334 differentially methylated genes (DMGs). Furthermore, GO/KEGG analyses showed that some DMGs were involved within immune response pathways. Combining WGBS-Seq data and existing RNA-Seq data, we identified 71 significantly differentially methylated (DMGs) and expressed (DEGs) genes (*p* < 0.05). Next, complementary analyses identified nine DMGs (*LTA*, *STAT3*, *IKBKG*, *IRAK1*, *NOD2*, *TLR2*, *TNFRSF1A*, and *IKBKB*) that might be involved in the immune response of XJB cattle infected with respiratory diseases. Although further investigations are needed to confirm their exact implication in the involved immune processes, these genes could potentially be used for a marker-assisted selection of animals resistant to BRD. This study also provides new knowledge regarding epigenetic control for the bovine respiratory immune process.

## 1. Introduction

Xinjiang brown cattle are local to the Xinjiang region of China, and their production represents an important part of Xinjiang’s agricultural sector and economy. However, bovine respiratory disease (BRD) is a serious threat to cattle survival in Xinjiang and the primary driver of antibiotic use. Clinical signs associated with BRD typically include elevated rectal temperature, increased respiratory rate, nasal and ocular discharges, cough, dyspnea, decreased appetite, and depression [1]. To reduce financial losses, reduce antibiotic use, and improve animal well-being, it is urgent to investigate some of the mechanism(s) underlying BRD. BRD not only kills cattle and leads to economic losses but is associated with higher treatment and fattening costs [2]. In addition, the higher use of antibiotics to treat the cattle may result in food safety issues.

BRD is considered a polymicrobial disease, which means that it arises from infections with a combination of bacteria and viruses [2]. Many efforts have been made to elucidate the mechanisms underlying calf susceptibility to respiratory diseases at different levels. For example, Keele et al. (2015) conducted a genome-wide association study of the presence/absence of lung lesions in cattle using sample pooling to identify important gene regions. Fourteen SNPs on BTA 2, 3, 4, 9, 11, 14, 15, 22, 24, and 25 were significant at the experiment-wise error rate of 5% (*p* ≤ 1.49 × 10^−7^) [3]. To date, the mechanisms underlying calf susceptibility to respiratory disease have been studied at the level of signal pathways, transcription, and translation, including negative regulation of the cellular protein metabolic process (GO:0031324) and the integral component of the plasma membrane (GO:0005887) [3,4,5]. In addition, emerging studies have demonstrated that epigenetics plays an important role in the modulation of natural immunity and susceptibility to diseases, especially in terms of DNA methylation [6,7,8]. However, the related epigenetic regulatory mechanisms are still poorly understood.

DNA methylation is an epigenetic regulation mechanism that orchestrates a variety of physiological activities, including organism maturation and gene imprinting. Epigenetic regulations also affect immune processes: variation in the methylation levels may be directly related to several cytokine expression profiles. For example, hypomethylation of *IL-6* and *IL-8* gene promoters is associated with cytokine production and rheumatoid arthritis [9], and DNA methylation causes changes in the expression of *TLR2*, *MD-2*, and *I-κBα*, resulting in different responses to lipopolysaccharide stimulation [10]. Such investigations support the hypothesis that DNA methylation could play a pivotal role in disease susceptibility and immunological responses. Seutter et al. (2020), using a fibroblast model, demonstrated that DNA methylation was associated with *IL-8* response after lipopolysaccharide (LPS) invasion of fibroblasts isolated from the same animal aged 5 and 16 months [11]. Wang et al. (2010) found that the methylation level of the *CD4* gene promoter segment in whole blood of dairy cows with mastitis was 16% higher than that in healthy dairy cows [12]. In addition, upregulated promoter methylation was linked to *CD4* downregulation, possibly due to the methyl group inhibiting the binding of transcription factors in affected animals. In an experimental mastitis model, *E. coli* infection induced promoter methylation loss and higher expression of the *TLR2* gene [13]. In DNA methylation studies, obvious sources of sample-wise biological variance are gender and cellular composition. In females, one of the X chromosome copies is inactivated [14] and there are also imprinted regions and other DNA gender-associated methylation differences on the autosomes [15]. Methylation changes at these sites were strongly associated with chronological age; however, for some sites methylation increased with age, while it decreased with age at others [16]. Therefore, we selected calves of identical age and gender for our research.

However, little is known about the expression profiles and the possible importance of DNA methylation in the susceptibility of Xinjiang brown cattle to respiratory diseases. The early immune system of calves, which depends heavily on antibodies in colostrum, is not fully developed, but significant differences in innate immunity exist in the early stage of life, with the immune processes being carried out in the whole-body blood [17]. In these processes, in-depth knowledge is of paramount importance regarding dynamic changes in the DNA methylation map within bovine blood. In this respect, the use of whole-genome bisulfite sequencing (WGBS), a highly specific and sensitive technique for single-base resolution analysis of the methylation pattern, should be of great interest [18]. Our research hypothesizes that DNA methylation of specific genes in calves affects gene expression, which subsequently influences the levels of innate immunity. In this study, we obtained the genomic methylation pattern of Xinjiang brown cattle affected by respiratory disease. Such results will provide new avenues to investigate the resistance mechanisms to this disease in that population, a potentially important step towards obtaining disease-resistant cattle.

## 2. Results

### 2.1. Genome-Wide DNA Methylation Profile Analyses

Global DNA methylation analysis of peripheral blood was conducted using WGBS with 20× genome coverage and a bisulfite conversion rate >99%. Overall, we obtained 78.26 Gb and 75.46 Gb of raw data for controls and cases, respectively. After excluding the low-quality data, we obtained around 280,000,000 clean reads with a Q30 range of 88.91–92.59% and a mapping rate of 70.42–73.19% for further analysis (Table 1).

**Figure 1:**
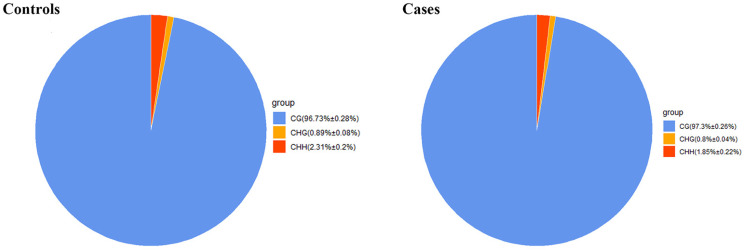
Average ratio of DNA methylation types in case and control genomes of Xinjiang brown cattle. Blue, orange, and red colors represent methylated (m) CG, mCHG, and mCHH, respectively.

**Figure 2:**
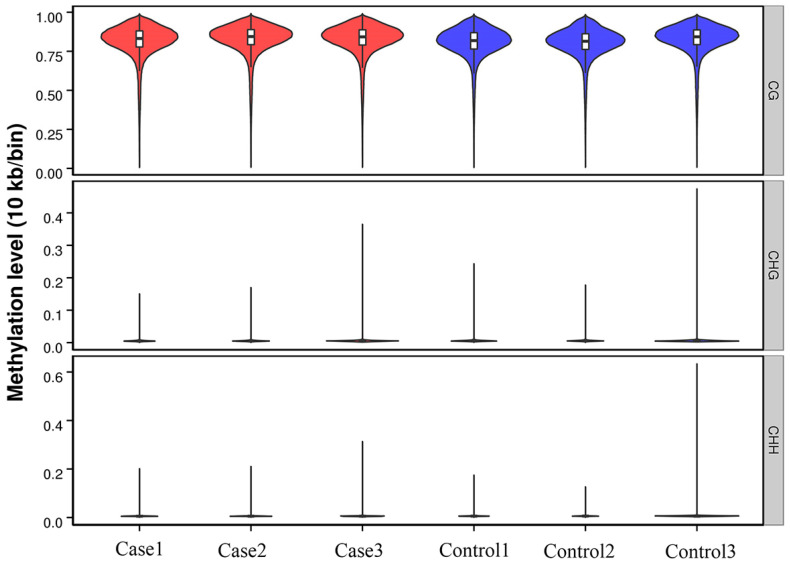
Violin plot of the overall status spread for the different methylation formats CG, CHG, and CHH. Controls (Control1, Control2, Control3) and cases (Case1, Case2, Case3). H = A, C, or T. Abscissas reflect individual samples; ordinates reflect methylation levels of individual samples. Violin width reflects datapoint density within a specific methylation level. Boxplot shows individual violin containing methylation levels. Red, Case group; Blue, Control group.

The methylated genomic C locations ranged from 3.05% to 3.39% among samples (Table 1). The methylation levels of CG, CHH, and CHG (whereby H = A, C, or T) differed: the methylated cytosine (mC) levels were 96.73 ± 0.28% for CG, 0.89 ± 0.08% for CHG, and 2.31 ± 0.2% for CHH in control samples, and 97.3 ±0.26% for CG, 0.8 ± 0.04% for CHG, and 1.85 ± 0.22% for CHH in case samples (Figure 1). Clearly, methylation levels were higher in CG than in CHG or CHH (Figure 2).

**Figure 3:**
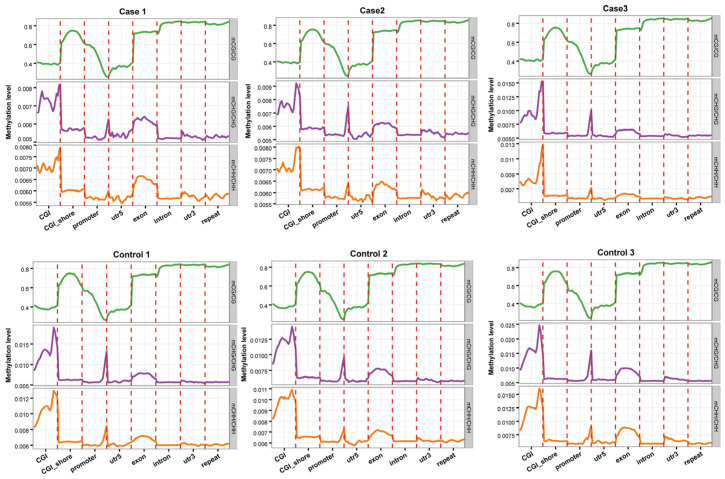
Distribution of methylation levels among different genomic elements. Abscissas represent different genomic elements and ordinates represent methylation levels. Each functional region of each gene is equally divided into 20 bins, and then the C-site level of the corresponding bin of the functional region of all genes is averaged. Different colors represent different sequence contexts (CPG, CHG, CHH).

To perform comparative analyses of the averaged methylation levels of the genomic regions in the two groups, we analyzed the methylation states of eight different segments, including CGI, CGI shores, promoters, 5′UTR, exons, introns, 3′UTR, and repeat segments. We visually observed no major variations in methylation levels between the case and control groups. DNA methylation levels in the CG environment were the highest for introns and the 3′UTR segments, followed by repeat segments and exons; the levels near the transcription initiation site (TSS) were the lowest. Methylation levels gradually decreased from the promoter to the TSS, and then gradually increased from the TSS to the intron (Figure 3).

### 2.2. DMR Profiling

**Figure 4:**
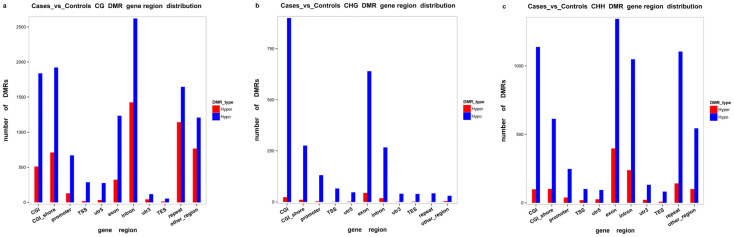
Identification of differentially methylated regions (DMRs) between case and control groups. Histograms show the distributions of DMRs in different genomic elements in the CG (**a**), CHG (**b**), and CHH (**c**) contexts. Hyper: hypermethylated genes, meaning that cases are more methylated than controls; Hypo: hypomethylated genes, meaning that cases are less methylated than controls.

**Figure 5:**
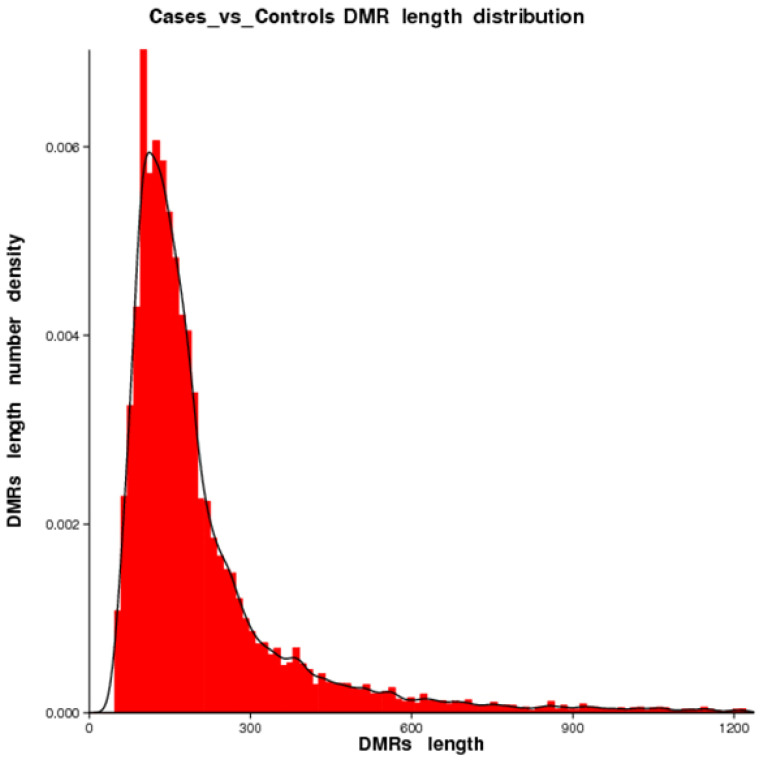
Distribution of DMR lengths.

We identified 16,765 DMRs for CG, 7502 DMRs for CHG, and 2656 for CHH. Hypermethylation (in which regions in cases are more methylated than in controls) occurred in 6269 (CG: 79.87%, CHH: 18.33%, CHG: 1.80%) and hypomethylation (in which regions in cases are less methylated than in controls) occurred in 20,654 DMRs (CG: 56.93%, CHH: 30.76%, CHG: 12.31%). DMRs were mostly located in introns, followed by exons, promoter regions, and the 5′UTR and 3′UTR segments. For CG, only 314, 159, and 857 DMRs were located in the 5′UTR, 3′UTR, and promoter segments, respectively. Figure 4 shows more details on the distribution of the DMRs. The length of DMRs ranged between 51 nt and 3332 nt (Figure 5).

### 2.3. GO/KEGG Enrichment Analysis for DMGs

**Figure 6:**
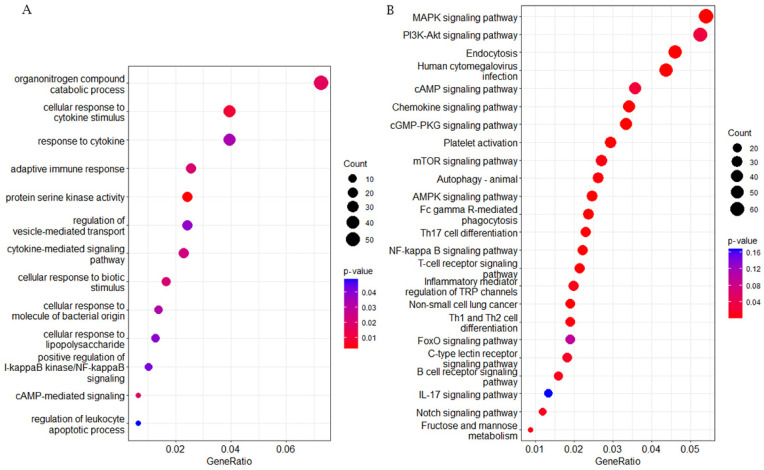
Enrichment analysis of CG-type differentially methylated genes (DMGs). (**A**) DMG enrichment scores for 13 peak-ranking immune response GO terms. Abscissas reflect genomic ratios (enrichment gene count/total gene count), while ordinates reflect GO pathway terms. (**B**) Scatterplot for 20 immune response KEGG pathways. Counts: enrichment gene count. *p*-value: corrected *p*-value, when testing the null hypothesis of no enrichment.

To probe changes in gene methylation state related to immunity to respiratory disease in brown cattle, 4334 DMGs—i.e., genes overlapping at least one DMR—were annotated to the GO and KEGG databases. The 13 most significant DMG-enriched pathways in GO were mostly related to immunity-linked terms (Figure 6A). Significant GO terms included upregulated I-kappa B kinase/NF-κappaB signal transduction, adaptive immune response, regulation of vesicle-mediated transport, and protein serine kinase activity. The 20 most significant DMG-enriched KEGG terms were in pathways related to immunity and had a corrected *p* < 0.05 (Figure 6B). These pathways included the T-cell receptor signaling pathway, platelet activation, fructose and mannose metabolism, Fc gamma R-driven phagocytosis, and the AMPK/NF-κappa B signaling pathways. In summary, DMGs might be related to immune function in calves. It is important to note that Kanehisa laboratories own the copyrights on these KEGG pathways [19].

### 2.4. Screening of Possibly Valuable DMGs Linked to Immune Function

**Figure 7:**
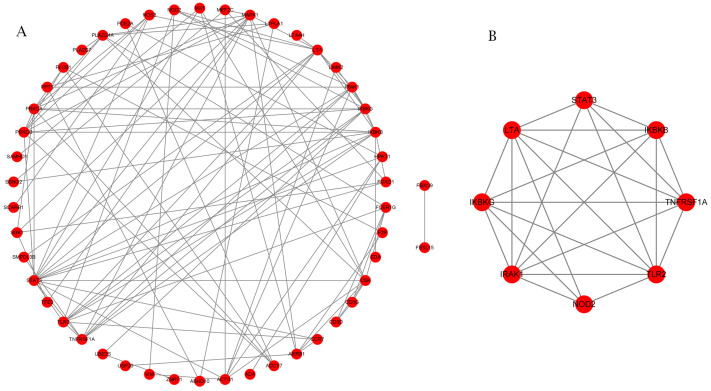
(**A**) Constructed DMG network linked with immune response. Evaluations of interplays among DMGs linked with immune response employing STRING^®^, in line with interplay index (confidence > 0.7). (**B**) Using the MCODE plug-in, the cluster with the highest score was selected to construct the relevant protein network diagram.

In an attempt to determine the pivotal genes linked to the immune control of BRD, we first identified 121 candidate genes linked to immunity based on GO and KEGG analyses. Among these candidate genes, 71 were present in the intersection of the DMGs and DEGs described above. Using STRING (v11.5) [20], we discovered 48 candidate genes with internal interactions. Using MCODE plug-in results within Cytoscape [21], we report the known interactions of these genes in Figure 7A, while the interactions of *LTA*, *STAT3*, *IKBKG*, *IRAK1*, *NOD2*, *TLR2*, *TNFRSF1A*, and *IKBKB*, eight genes classified by MCODE as hub genes within the interactions network linked to immune response pathways, are shown in Figure 7B.

### 2.5. Differential Gene Methylation Regulating Influence upon the Immunology of Cattle

**Figure 8:**
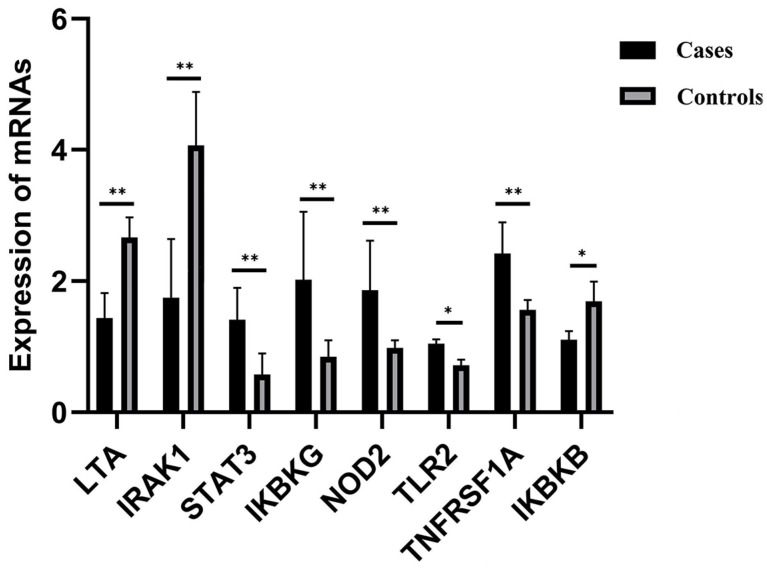
RT-qPCR was conducted to determine relative quantified transcriptomic expression for blood-borne candidate genes. *GAPDH* served as a normalization/reference gene. Values reflect mean ± SEM for three replicates. * *p* < 0.05, ** *p* < 0.01.

FPKM values of RNA-seq data were used to compare trends of genomic expression and methylation profiles between the case and control groups for these eight hub genes. DMRs for the *LTA* gene in exons, 5′UTR, TSS, and promoters and for the *IRAK1* gene in exons, introns, 5′UTR, TSS, and promoters were hypermethylated in cases with respect to controls while the corresponding transcribed regions were underexpressed. Conversely, hypomethylation was observed in cases compared to controls for the *STAT3* gene in introns, the *IKBKG* gene in TSS, exons, 5′UTR, introns, and promoters, the *NOD2* and *TLR2* genes in exons, and the *TNFRSF1A* gene in the promoter, introns, exons, and 3′UTR, while these genes were overexpressed in case groups. *IKBKB* introns were hypomethylated in cases with respect to controls, while this gene was underexpressed. RT-qPCR results confirmed the RNA-seq analyses. The DNA methylation patterns in the *LTA*, *IRAK1*, *IKBKG*, and *TNFRSF1A* promoter regions seem to have influenced the gene expression profiles, while the influence of the DNA methylation profile of alternative regions on the expression of the corresponding genes *STAT3*, *NOD2*, *TLR2*, and *IKBKB* is uncertain (*p* > 0.01; see Table 2 and Figure 8).

## 3. Discussion

DNA methylation is an important mechanism in epigenomic control which plays a pivotal role in genomic expression/tissue development [22]. Although DNA methylation in the muscle tissue of cattle [23], pigs [24], humans [25], mice [22], and sheep [26] has already been analyzed, whole-genome DNA methylation analysis of bovine blood is relatively scarce. To the best of our knowledge, this study has pioneered a systematic comparative analysis method for genomic DNA methylation patterns in Xinjiang brown cattle affected or not affected by a respiratory disease. In previous studies, transcriptome analysis confirmed that many immune-related genes and KEGG pathways were differentially expressed between affected and non-affected groups [27]. Therefore, we employed WGBS technology to study DNA methylation in the bovine blood genome to clarify the relationship between respiratory immune differences and DNA methylation.

In the tested genes’ functional regions, genome-wide methylation profiles were rather similar between the case and control groups (Figure 3). However, variations existed between the three mC contexts (i.e., CG, CHG, and CHH), with differences between the various genomic regions [28]. Out of the 280,000,000 clean reads obtained using WGBS-seq, we could map 70.42–73.19% of the reads to unique positions, which was less than bovine skeletal muscle satellite cells through MeDIP [23]. About 3.17% of all cytosine sites showed methylation, with a vast majority in CG groups (Figure 1 and Figure 2), in line with reports on other organisms [29]. In the gene functional regions, the methylation level of TSS was lowest, consistent with Zhang et al. (2017) [30]. We identified 26,923 DMRs and 4334 DMR-correlated genes. DMRs were mainly located in the promoter, 3′UTR, and 5′UTR regions of genes, where they only accounted for a small part of these regions.

GO analyses for DMGs pointed to several important pathways related to immunity, as summarized above, and KEGG analyses further validated the results. The activation of Toll-like receptors (TLRs) induces the NF-kappaB signaling pathway, thus activating an inflammatory response [31]. The NF-kappa B signaling pathway is very important for the immune recognition of LPS and the release of inflammatory factors [32]. When the AMPK signaling pathway is activated, it can improve cell lipid metabolism that alleviates inflammation by regulating cellular lipid metabolism enzymes and autophagy. AMPK helps host cells resist pathogen invasion by inducing the expression of autophagy-related genes and promoting phagosome maturation. In addition, in immune cells, activated AMPK plays an anti-inflammatory role by directly promoting inflammatory signals and inhibiting the synthesis of some lipid intermediates related to inflammation [33]. In our study, DMGs linked to such activities showed major differences between case and control groups, indicating possibly pivotal roles in immune recognition. Since little is known about the mechanisms underlying DNA methylation and how this may influence genomic expression, we performed a transcriptomic analysis of the DMG to evaluate the effect on the expression of these genes. This allowed us to identify genes with an altered expression between cases and controls (DEGs).

We established a DMG interactivity network to find out whether DMGs were playing pivotal parts within bovine immune roles. Networking analyses showed *LTA*, *STAT3*, *IKBKG*, *CSK*, *IRAK1*, *NOD2*, *TLR2*, *TNFRSF1A*, and *IKBKB* were the key nodes. The *LTA* gene encodes tumor necrosis factor beta cytokine, which is vital for regular immune maturation. Within *LTA* knockout mice, all Peyer’s patches/lymph nodes did not mature, suggesting the importance of *LTA* for such immune activities [34]. *STAT3* is an acute-phase responsive factor that triggers *IL-6* [35]. Its overexpression reduces the severity of inflammation in mice [36] and orchestrates Th17 cellular maturation/cytokine discharge through Th2/Th17, contributing to asthma [37]. *IKBKG* binds to the Ripk3 promoter, inhibiting its transcription. Ripk3 promotes NF-κB signal transduction, an important pathway of the inflammatory response [38]. *CSK* downregulates T-cell antigen receptor (TCR)-driven signal transduction [39], resulting in thwarting of Lck-driven ζ chain phosphorylation, thus preventing proximal T-cell triggering [40]. *IRAK1* is a downstream effector for TLR signaling pathways, with inflammatory, autoimmune, and cancer-linked activities [41]. Restoring *IRAK1* expression reverses the effects of miR-146b-5p on EGFR TKI sensitivity and recuperates NF-κB-controlled IL-6 and IL-8 discharge [42]. NF-κB signal transduction can be avoided by degrading IRAK1 [43].

*NOD2* is key for immediate innate immune clearing-up for Acinetobacter baumannii within lungs [44]. It also enhances clearing-up for Chlamydophila pneumonia within lungs [45] and orchestrates M. tuberculosis immunity [46,47]. *TLR2* stimulation inducible protein (IP)-10 leads to discharging of the IL-12-thwarting p40 homodimer, creating a favorable environment for Th2 progression. *TLR2* triggering additionally leads to skewed triggering for IL-8 and IL-23 [48]. TLR2-driven innate immune priming enhances pulmonary anti-viral immune resilience [49]. In addition, *TLR4* jointly regulates the NF-κB signaling pathway and affects immune factors. *TNFRSF1A* encodes *TNFR1* and suppresses the expression of inflammatory cytokines [50]. It is an essential orchestrator of TNFα-driven NF-κB function [51]. Multiple signaling transduction pathways triggering NF-kB meet up at the *IKKB* level [52].

Although the role of the detected genes in immunity has been demonstrated, knowledge is scarce regarding the role of methylation in *LTA*, *STAT3*, *IKBKG*, and *IRAK1* and how it may regulate mammalian immunity. Some investigations have demonstrated versatile shifts in DNA methylation profiles across cellular differentiation procedures [53,54], suggesting that such genes have pivotal roles in immunity maturation. This could explain immune variations between cases and control groups.

In summary, DNA methylation can influence immune function in cattle. There is an intricate interplay between DNA methylation and genomic expression profiles [55]. Even though DNA methylation in a promoter region can thwart genomic expression [56], knowledge on the mechanisms by which DNA methylation influences the latter expression is still lacking [57]. In our study, we found that hypermethylation in the promoter region thwarted *IKBKG* and *TNFRSF1A* expression, while hypomethylation in the promoter region upregulated *LTA* and *IRAK1* expression.

In the present study, the cattle were infected by bacteria. The immune system of newborn calves is imperfect; innate immunity is crucial in combating early infections. Bacterial infection quickly activates the innate immune response and triggers inflammation in order to enable host defense. We conducted an analysis to assess the relationship between alterations in DNA methylation patterns and corresponding changes in gene expression levels. It was observed that variations in gene expression were linked to modifications in methylation patterns. Variations in DNA methylation levels of immune-related genes contribute to differential expression patterns, ultimately influencing the strength and rate of development of the calf’s innate immune system. These variations impact the calf’s ability to recognize bacteria, thereby affecting the speed and intensity of immune response and subsequently influencing immunity to pneumonia. Networking analyses revealed nine key genes, suggesting that these nine key genes had close interaction and together determined the key pathway during the innate immune response. Functional and pathway enrichment analysis of genes mainly involved the NF-kappa B signaling pathway. Studies have demonstrated that the NF-kappa B signaling pathway plays an important role in innate immune response [32]. Thus, our study suggests that this pathway in immunity might represent a promising candidate for developing innate immunity in calves. *IKBKG*, *TNFRSF1A*, *LTA*, and *IRAK1* are key regulatory genes in the immune process. Their methylation can partly lead to major variations in immunity, especially innate immunity. However, the epigenomic mechanisms underlying this process require additional research.

This study had several limitations. First, the study sample size is very small. Future studies with larger sample sizes are needed to replicate and corroborate our findings. Second, the use of blood and not tissues of the upper and lower respiratory tract limits the scope of the current work. These limitations will be further investigated in future research.

## 4. Materials and Methods

### 4.1. Animal Experiments

All experiments were performed in accordance with the guidelines established by the Animal Care Committee of Xinjiang Agricultural University (No. 2018017). Three diseased calves and three healthy calves were purchased from Altay, Xinjiang (Xinjiang, China). The calves, three-week-old females of similar weight, were raised in identical environments with unlimited access to food and water, the same nutritional levels, and communal housing. Blood samples were extracted from the jugular vein, flash-frozen in liquid nitrogen, and kept at −80 °C.

Diseased animals were confirmed as BRD-positive cases based on clinical examination and serum haptoglobin concentration, i.e., animals with at least one visual BRD sign, a rectal temperature ≥40 °C, abnormal lung sounds detected at auscultation, and a serum haptoglobin concentration ≥0.25 g/L. Healthy animals, showing no visual signs of BRD, with a rectal temperature <40 °C, with no abnormal lung sounds detected at auscultation, and with a serum haptoglobin concentration <0.25 g/L, were considered as control animals in the following analyses. Symptoms of clinical diagnosis include bilateral mucopurulent nasal discharge, heavy eye discharge, head tilt or droop, repeated coughing, high temperature, and watery diarrhea. At the same time, nasal swabs from cases and controls were collected for isolation and identification of pathogenic bacteria. We found that the selected positive individuals were infected by Pasteurella multicida type-A using PCR and sequencing, while we isolated no bacteria from the healthy animals.

### 4.2. Library Construction

Genomic DNA of the blood from the six calves was extracted using a TIANamp Genomic DNA Kit (Tiangen, Beijing, China). The DNA quantity and quality were determined using a NanoDrop2000 spectrophotometer (ThermoFisher, Waltham, MA, USA) and Agilent 2100 Bioanalyzer (Agilent, Santa Clara, CA, USA), respectively. 

First, 5 μg of lambda DNA was added into the genomic DNA of each sample as the negative control. Then, the genomic DNA was randomly broken into 200–300 bp fragments using an S220 focused-ultrasonicator (Covaris, Santa Clara, MA, USA); these fragments were end-repaired, added to the sequencing adapter, and treated with bisulfite. The methylated cytosines were thereby unchanged and the unmethylated cytosines became uracils, which were changed to thymines after PCR amplification. A library quality control was performed using an Agilent 2100 Bioanalyzer [58].

### 4.3. Whole-Genome Bisulfite Sequencing (WGBS) and Differentially Methylated Region (DMR) Determination

With the completion of the library, paired-end sequencing was performed on the Illumina HiSeqTM2500 sequencing platform (Illumina, San Diego, CA, USA). Preliminary quality control of raw reads was carried out with FastQC (v0.11.9, https://www.bioinformatics.babraham.ac.uk/projects/fastqc/, accessed on 6 May 2023), and these reads were then filtered with fastp software (v0.20) to remove adapters and low-quality sequences (i.e., sequences with phred scores below 30 in the paired-end 150 bp raw sequencing files [59]).

Clean reads were aligned to a cattle reference genome (ARS-UCD1.2, https://asia.ensembl.org/index.html, accessed on 6 May 2023), and bisulfite mapping of methylation sites was conducted using Bismark (v0.22.1) [60]. Duplicates consisted of reads aligning with identical genomic regions. The average number of duplicates over the whole genome is the sequencing depth/coverage. The bisulfite conversion rate was the percentage of reads with at least one methylated cytosine among the clean reads spanning that cytosine, averaged over the whole genome. We used a binomial test for individual C sites to confirm C-site methylation by screening positions with a coverage ≥4× and using a false discovery rate (FDR) <0.05.

To identify regions that were statistically differentially methylated between control and case samples (i.e., DMRs), we first defined genomic regions: promoter regions (2 kb upstream of the transcription start position of each gene), 5′UTR regions, exons, introns, 3′UTR regions, CGI regions, CGI shore regions, TSS regions, TES regions, repeat regions, and other (than the previous) regions. The methylation levels in these regions were evaluated using the model of Lister et al. (2011) [29]. Roughly, this model computes the methylation level of a tested region as the ratio mCG/(mCG + nmCG) in that region, where mCG stands for the number of methylated CpG groups and nmCG for the number of non-methylated CpG groups. We employed DSS analysis software for DMR (differentially methylated region) analyses [61].

### 4.4. Functional Enrichment Analysis

Gene ontology (GO) enrichment and Kyoto Encyclopedia of Genes and Genomes (KEGG) analyses were conducted using the ClusterProfiler (version 4.3.0, https://github.com/GuangchuangYu/clusterProfiler, accessed on 15 May 2023) package [62], and enrichments with adjusted *p*-values < 0.05 were deemed statistically significant. The STRING online repository was employed to predict protein–protein interactions (PPIs) (STRING; http://string-db.org, accessed on 17 May 2023) (version 10.0) [63]. Cytoscape (version 3.9) [64] and its plug-in Molecular Complex Detection (MCODE) (version 2.0.0) were employed to explore important differentially methylated genes (DMGs) [31].

### 4.5. Quantitative Reverse Transcription-PCR

Samples for RT-qPCR analysis were the same as those for WGBS. We tested DMGs for potential differential expression through RT-qPCR. Total RNA was extracted from blood with Trizol^®^ reagent (Invitrogen™, Waltham, MA, USA). cDNA was reverse transcribed from total RNA using the PrimeScript RT kit^®^ (Takara™, Beijing, China). RT-PCR was conducted using the StepOnePlus Real-Time PCR System^®^ (Life Technologies™, Waltham, MA, USA) with SYBR Green Master Mix (Roche Applied Science, Mannheim, Germany). Relative expression of individual genes was normalized to GAPDH using the 2^−ΔΔCt^ methodology [65]. Although the GAPDH gene has been widely used as a housekeeping gene in qPCR for a long time as a key gene in glycolysis, many factors can nevertheless affect its expression in different metabolic processes. In this study, almost all detected candidate genes were related to immunity (see below), which retrospectively makes GAPDH suitable as an internal reference.

### 4.6. RNA-Seq Data Analysis

We next performed a whole-genome transcriptome analysis. Samples for transcriptome analysis were the same as those for WGBS. Six cDNA libraries were sequenced by the Illumina NovaSeq 6000 (Novogene™, Beijing, China) sequencing platform. The raw data obtained by sequencing were subjected to quality evaluation using FastQC (v0.11.9, https://www.bioinformatics.babraham.ac.uk/projects/fastqc/, accessed on 17 March 2023), and the original sequence was washed and filtered using Seqtk (v1.2, https://github.com/lh3/seqtk, accessed on 17 March 2023) software, and the linker sequence, low-quality, and “N”-containing reads were filtered out. The filtered clean reads were compared to the Bos taurus reference genome using Hisat 2.0 (v2.0.1, https://daehwankimlab.github.io/hisat2/, accessed on 17 March 2023) software (ARS-UCD1.2). The expression level of each gene was normalized using edgeR (version 3.16.5, https://bioconductor.org/packages/release/bioc/html/edgeR.html, accessed on 17 March 2023) software to calculate the FPKM value. In this study, both *p* < 0.05 and |Log2FC| > 2 were taken as the thresholds for significant differential expression (i.e., DEGs: differentially expressed genes).

### 4.7. Correlation Analysis

For correlation analysis, a set of differentially expressed genes with differential methylation was obtained from the intersection between the set of differentially methylated genes (DMGs)—i.e., genes for which the promoter or the region from the TSS to the TES overlaps with a DMR—and the set of differentially expressed genes (DEGs). We next performed correlation analysis between the methylation level of DMRs and the expression level of the corresponding DEGs [55] and checked using GO or KEGG whether the most correlated genes were related.

## 5. Conclusions

We probed, in a pioneering, systematic manner, genome-wide DNA methylation profiles from blood samples of calves with anti-respiratory diseases and susceptible respiratory diseases, and investigated several novel and valuable DMRs/DMGs together with pathways related to bovine innate immune response. These dataset outcomes contributed essential information regarding possible in-depth knowledge gains on genomic/epigenomic mechanisms for bovine immune traits, for deployment as a biomarker-facilitated screening program for promoting bovine immunity.

## Figures and Tables

**Table 1:** Sequencing data by whole-genome bisulfite sequencing (WGBS).

Group	Samples	Clean Bases (Gb)	Clean Reads	GC (%)	Q 30 (%)	Mapping Rate (%)	Bs Conversion Rate (%)	mC Percent (%)
Cases	Case1	76.94	280,373,522	23.22	91.9	72.05	99.742	3.13
	Case2	78.94	287,142,601	22.86	92.59	72.12	99.717	3.21
	Case3	78.90	287,493,079	22.81	91.37	72.05	99.703	3.19
Controls	Control1	75.60	277,034,171	22.77	89.35	70.79	99.702	3.05
	Control2	74.03	272,716,162	22.73	88.91	70.42	99.673	3.07
	Control3	76.73	279,618,157	23.05	91.34	73.19	99.707	3.39

**Table 2:** DMGs putatively linked to immune difference.

RNA-Seq		WGBS-Seq		
Gene	Regulation	Meth Chr	Annotation	Stat
LTA	Underexpressed	23	exon, utr5, TSS, promoter,	hyper
IRAK1	Underexpressed	X	exon, intron, utr5, TSS promoter	hyper
CSK	Underexpressed	21	intron, exon, utr5	hyper
STAT3	Overexpressed	19	intron	hypo
IKBKG	Overexpressed	X	TSS, exon, utr5, intron, promoter	hypo
NOD2	Overexpressed	18	exon	hypo
TLR2	Overexpressed	17	exon	hypo
TNFRSF1A	Overexpressed	5	promoter, intron, exon, utr3	hypo
IKBKB	Underexpressed	27	intron	hypo

## Data Availability

The RNA sequencing and WGBS data were deposited at NCBI Short Reads Archive (SRA), and the project accession numbers are PRJNA702464 and PRJNA861721.
